# Can arbuscular mycorrhizal fungi reduce Cd uptake and alleviate Cd toxicity of *Lonicera japonica* grown in Cd-added soils?

**DOI:** 10.1038/srep21805

**Published:** 2016-02-19

**Authors:** Qiu-Yun Jiang, Feng Zhuo, Shi-Hui Long, Hai-Di Zhao, Dan-Jing Yang, Zhi-Hong Ye, Shao-Shan Li, Yuan-Xiao Jing

**Affiliations:** 1 Key Laboratory of Ecology and Environmental Science in Guangdong Higher Education, Guangzhou Key Laboratory of Subtropical Biodiversity and Biomonitoring, College of Life Sciences, South China Normal University, Guangzhou 510631, P. R. China; 2 Guangzhou Research Academy of Environmental Protection, Guangzhou 510620, P. R. China; 3 State Key Laboratory for Bio-control, and School of Life Sciences, Sun Yat-sen University, Guangzhou 510006, P. R. China

## Abstract

A greenhouse pot experiment was conducted to study the impact of arbuscular mycorrhizal fungi−*Glomus versiforme* (Gv) and *Rhizophagus intraradices* (Ri) on the growth, Cd uptake, antioxidant indices [glutathione reductase (GR), ascorbate peroxidase (APX), superoxide dismutase (SOD), catalase (CAT), ascorbate (ASA), glutathione (GSH) and malonaldehyde (MDA)] and phytochelatins (PCs) production of *Lonicera japonica* in Cd-amended soils. Gv and Ri significantly increased P acquisition, biomass of shoots and roots at all Cd treatments. Gv significantly decreased Cd concentrations in shoots and roots, and Ri also obviously reduced Cd concentrations in shoots but increased Cd concentrations in roots. Meanwhile, activities of CAT, APX and GR, and contents of ASA and PCs were remarkably higher in Gv/Ri-inoculated plants than those of uninoculated plants, but lower MDA and GSH contents in Gv/Ri-inoculated plants were found. In conclusion, Gv and Ri symbiosis alleviated Cd toxicity of *L. japonica* through the decline of shoot Cd concentrations and the improvement of P nutrition, PCs content and activities of GR, CAT, APX in inoculated plants, and then improved plant growth. The decrease of shoot Cd concentrations in *L. japonica* inoculated with Gv/Ri would provide a clue for safe production of this plant from Cd-contaminated soils.

Cadmium (Cd) is a potentially hazardous heavy metal (HM) that can be phytotoxic even at very low level (0.5 μg Cd g^−1^ soil)^1^,^2^. Cd can induce generation of reactive oxygen species (ROS) causing lipid peroxidation and, consequently, increasing membranes permeability^3^,^4^. To counteract oxidative stress, there are enzymatic and non-enzymatic defense systems in plants, including the dismutation of O_2_^−^ to H_2_O_2_ and O_2_ by superoxide dismutase (SOD)^5^, the detoxification of H_2_O_2_ by peroxidase (POD), catalase (CAT) and ascorbate peroxidase (APX)^6^,^7^, the catalysis of oxidized glutathione to reduced glutathione (GSH) by glutathione reductase (GR)^8^, and the ascorbate-glutathione pathway. Moreover, phytochelatins (PCs) product also has been regarded as an important detoxifying mechanism for HMs in plants^9^. Formed PCs-metal complexes can participate in HM detoxification and/or HM allocation among different plant organs^10^.

Human activities such as smelting, mining, plating and extensive chemical use in agriculture, cause HM-contamination in the soils throughout the world^11^. HM can accumulate in the edible plant tissues at rates sufficient to cause a threat to human health^12^. To reduce toxic HM concentrations in plants, some strategies have been put forward. Of these, microorganisms closely associated with plant roots would be preferred, since they can influence plant growth and metal uptake in environmental friendly ways^13^,^14^.

Arbuscular mycorrhizal fungi (AMF) can form symbiotic relations with the roots of most terrestrial plant species^15^. AMF can stimulate plant growth by increasing nutrient uptake^16^,^17^, supply a HM isolation belt to protect plant from HM toxicity, and reduce the absorption of HM by plants through secreting organic compounds (e.g., glomalin) in the soil to chelate HM ions^18^,^19^. Moreover, AMF could increase the activities of antioxidative system under HM stress^20^,^21^, and some genes with presumptive roles in alleviating oxidative stress were found in AMF^22^. However, the overall mechanisms by which AMF alleviate HM phytotoxicity have still been not completely understood, with controversial outcomes depending on the interactions of specific plant, fungus and HM species.

*Lonicera japonica* Thunb., a medicinal and an ornamental plant for vertical gardening, has been widely planted in temperate and tropical regions in the past 150 years^23^. It possesses many characteristics, such as high biomass, deep root, easy cultivation, wide geographic distribution and strong resistance to environmental stress^24^. Recently, Liu *et al.* and Jia *et al.* have found that *L. japonica* had a strong capability in Cd accumulation, which would bring out a threat to safe production of this plant^25^,^26^,^27^. So far, no information has been available on the role of AMF in Cd uptake and Cd toxicity relief in *L. japonica*. In this study, we explored whether AMF-*Glomus versiforme* (Gv) and *Rhizophagus intraradices* (Ri) could reduce Cd uptake and alleviate Cd phytotoxicity in *L. japonica* planted in Cd-amended soils (0, 10 and 20 μg Cd g^−1^), and further provided an enlightenment to the mechanism of Cd toxicity relief in mycorrhizal plant through determining plants biomass, Cd concentration, antioxidant activities and PCs production in plants.

## Results

Two-way ANOVA with AMF inoculation, Cd addition and their interaction were shown in Table 1. Each of the two factors separately generated significant differences in all variables, with the exception of SOD activity and soil DTPA-Cd concentration (AMF inoculation) and shoot biomass (Cd addition). Moreover, the Cd × AMF interaction generated significant changes in both shoot and root Cd concentrations and PCs content, and also resulted in evident changes in all antioxidative parameters, with the exception of SOD activity and GSH content (Table 1).

### Mycorrhizal colonization rate

**Figure 1:**
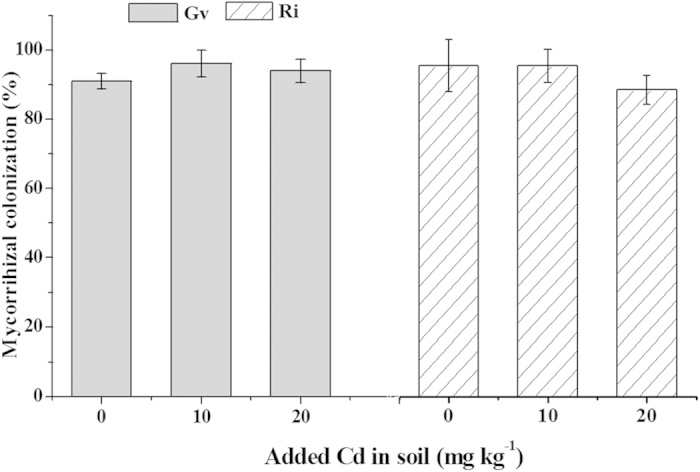
Mycorrhizal colonization rates of *L. japonica.* Gv, *Glomus versiforme*; Ri, *Rhizophagus intraradices*. Values are presented as means ± SD for the five replicates. An asterisk (*) within each arbuscular mycorrhizal fungus denotes that there is a significant difference between Cd-added and Cd-unadded soils according to the Tukey test at the 5% level.

Mycorrhizal colonization of *L. japonica* was observed in the all inoculation groups (Fig. 1). Mycorrhizal colonization rates of *L. japonica* were quite high, from 91% to 96% for Gv and from 89% to 96% for Ri, respectively. Compared with the Cd-unadded soil, mycorrhizal colonization rate was hardly affected by Cd addition. Moreover, hyphae or vesicles were not found in the uninoculation controls.

### Plant growth and P acquisition

**Figure 2:**
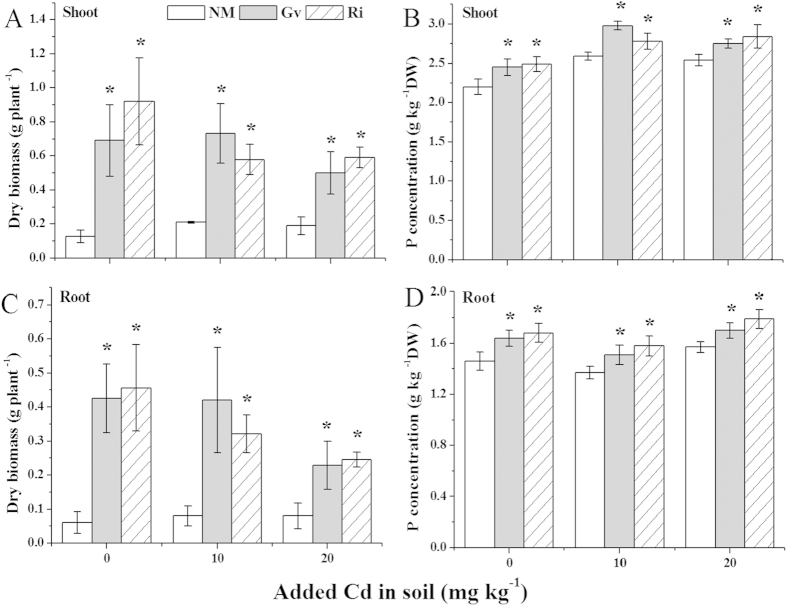
Dry biomass of the shoot (**A**) and root (**C**), P concentration in the shoot (**B**) and root (**D**) of *L. japonica*. Gv, *Glomus versiforme*; Ri, *Rhizophagus intraradices*; NM, Non-mycorrhiza. Values are presented as means ± SD for the five replicates. An asterisk (*) within each Cd concentration denotes that there is a significant difference between inoculation and uninoculation treatments according to the Tukey test at the 5% level.

The positive effect of both Gv and Ri inoculations on the dry weight and P acquisition of the shoot and root of *L. japonica* at the all Cd levels were shown in Fig. 2. The biomass of *L. japonica* inoculated with AMF were significantly (*P* < 0.05) elevated in the soils added with 0, 10, 20 μg Cd g^−1^, with the increases of 444%, 248%, 163% for Gv and 625%, 176%, 212% for Ri in the shoots (Fig. 2A), and 598%, 425%, 186% for Gv and 648%, 301%, 206% for Ri in the roots (Fig. 2C), respectively, compared with the uninoculation control. Similarly, the evident increases (*P* < 0.05) of P concentrations in mycorrhizal *L. japonica* in 0, 10, 20 μg Cd g^−1^ soils, were also observed, which were 11%, 15%, 8% for Gv and 13%, 7%, 12% for Ri in the shoots (Fig. 2B), and 12%, 10%, 8% for Gv and 15%, 15%, 14% for Ri in the roots (Fig. 2D), respectively.

### Plant Cd concentrations and soil DTPA-extractable Cd

**Figure 3:**
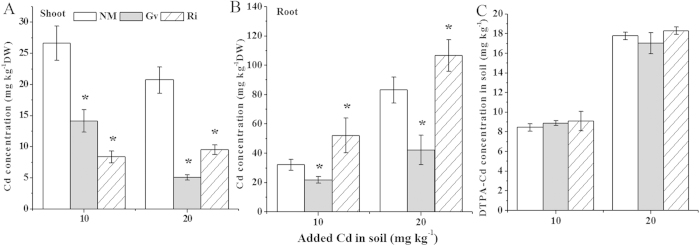
Cd concentration in shoot (**A**) and root (**B**) of *L. japonica*, soil DTPA-extractable Cd concentration (**C**). Gv, *Glomus versiforme*; Ri, *Rhizophagus intraradices*; NM, Non-mycorrhiza. Values are presented as means ± SD for the five replicates. An asterisk (*) within each Cd concentration denotes that there is a significant difference between inoculation and uninoculation treatments according to the Tukey test at the 5% level.

AMF inoculations significantly (*P* < 0.05) influenced Cd concentrations in the shoots and roots of *L. japonica* (Fig. 3). Compared with uninoculation groups, Cd concentrations in plants inoculated with Gv in 10 and 20 μg Cd g^−1^ soil were markedly (*P* < 0.05) reduced by 47% and 76% in the shoots (Fig. 3A) and 32% and 49% in the roots (Fig. 3B), respectively. Furthermore, Ri inoculation evidently (*P* < 0.05) reduced Cd concentrations in the shoots, with the reductions of 69% and 54% (Fig. 3A), but obviously (*P* < 0.05) improved Cd concentrations in the roots, with the increases of 62% to 28% (Fig. 3B) in 10 and 20 μg Cd g^−1^ soil, respectively. Moreover, soil DTPA-extractable Cd concentrations in 10 and 20 μg Cd g^−1^ soils were not obviously affected by both Gv and Ri inoculations (Fig. 3C).

### Antioxidative parameters and lipid peroxidation

**Figure 4:**
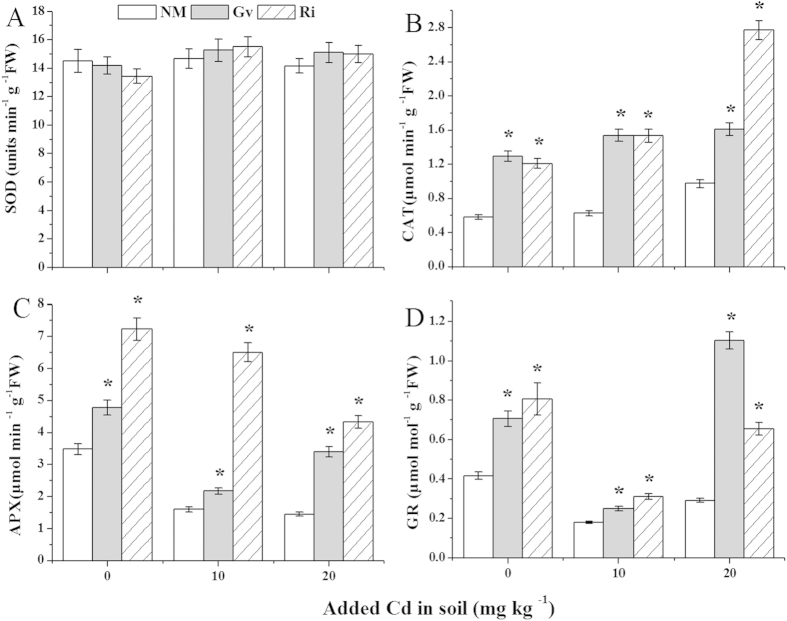
Activities of superoxide dismutase (SOD) (**A**), catalase (CAT) (**B**), ascorbate peroxidase (APX) (**C**) and glutathione reductase (GR) (**D**) in the leaves of *L. japonica.* Gv, *Glomus versiforme*; Ri, *Rhizophagus intraradices*; NM, Non-mycorrhiza. An asterisk (*) within each Cd concentration denotes that there is a significant difference between inoculation and uninoculation treatments according to the Tukey test at the 5% level.

**Figure 5:**
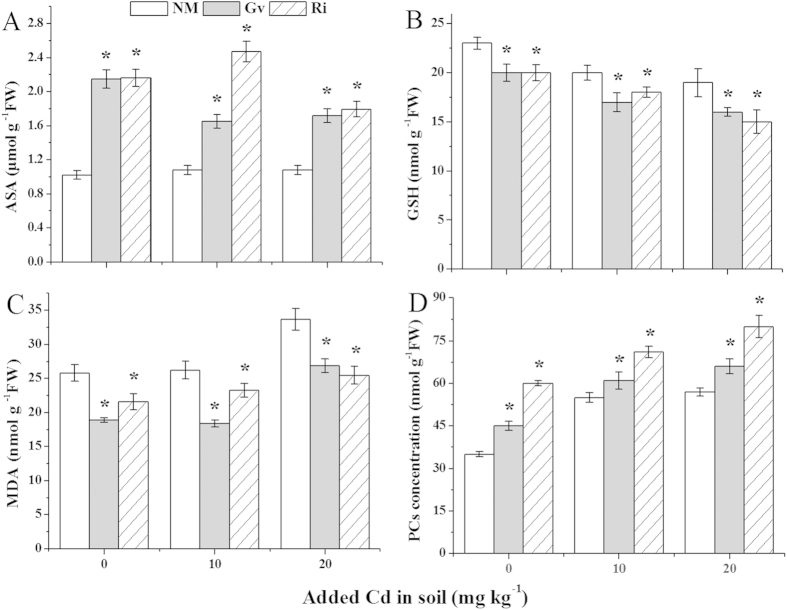
Contents of ascorbate (ASA) (**A**), reduced glutathione (GSH) (**B**), malonaldehyde (MDA) (**C**) and phytochelatins (PCs) (**D**) in the leaves of *L. japonica.* Gv, *Glomus versiforme*; Ri, *Rhizophagus intraradices*; NM, Non-mycorrhiza. An asterisk (*) within each Cd concentration denotes that there is a significant difference between inoculation and uninoculation treatments according to the Tukey test at the 5% level.

The parameters of antioxidant activities in *L. japonica* with and without AMF were presented in Figs 4 and 5. Compared with the uninoculated plants, AMF colonization markedly increased (*P* < 0.05) the activities of CAT, APX and GR in mycorrhizal plants at the all Cd levels, with the increases from 65% to 146% for Gv and 107% to 184% for Ri in CAT (Fig. 4B), 36% to 134% for Gv and 108% to 305% for Ri in APX (Fig. 4C), and 39% to 278% for Gv and 74% to 124% for Ri in GR (Fig. 4D), respectively. However, both Gv and Ri colonization had no impact on SOD activities at the all Cd levels (Fig. 4A).

Acting as antioxidants, the ASA contents in mycorrhizal plants were obviously (*P* < 0.05) increased, with the increases from 53% to 110% for Gv and 66% to 128% for Ri (Fig. 5A), but the GSH contents in mycorrhizal plants were evidently (*P* < 0.05) decreased, with the decreases from 13% to 16% for Gv and 10% to 21% for Ri, respectively (Fig. 5B), compared with non-mycorrhizal plants at all tested Cd levels. In addition, as an indicator of lipid peroxidation, MDA contents in inoculated plants had a pronounced (*P* < 0.05) decrease at all Cd levels, with the decreases from 20% to 30% for Gv and 11% to 24% for Ri (Fig. 5C), compared with uninocualted controls.

### Phytochelatins

The PCs contents in *L. japonica* with and without AMF were observed (Fig. 5D). The presence of AMF obviously (*P* < 0.05) increased the PCs production in mycorrhizal plants at all Cd levels, with the increases from 11% to 29% for Gv and 29% to 71% for Ri, respectively, compared with non-inoculated plants.

## Discussion

Previous studies have indicated that Cd addition in the soil did not inhibit the formation of external hyphae and mycorrhizal colonization. For example, Chen *et al.* found that soil Cd contaminations (0 to 100 μg g^−1^) did not affect *Funneliformis mosseae* colonization to *Zea mays*^18^, and Jiang *et al.* also discovered that *F. mosseae* colonization to *Solanum nigrum* remained unaffected in 0–40 μg Cd g^−1^ soil^28^, which are in accord with our present outcomes. The present results displayed that both Gv and Ri presented high resistance to Cd and could well associate with *L. japonica*.

Cd stress could inhibit roots growth and nutrition absorption especially P, thus affected the whole plant growth^29^,^30^. However, AMF may improve nutritional status and plant growth by the large surface area of their hyphae^31^. In the present work, the P absorption and biomass were observably increased in the shoot and root of *L. japonica* with both Gv and Ri inoculation. These results were similar to previous findings^10^,^32^,^33^, in which AMF colonization promoted P acquisition and plant growth.

Some studies have reported that AMF could immobilize HMs in the mycorrhizosphere and inhibit their translocation to the shoots. For instance, Bissonnette *et al.* reported that *Rhizophagus intraradices* inoculation in *Salix viminalis* reduced Cd concentrations in the shoots, but increased Cd concentrations in the roots^34^. Similarly, our previous study also indicated that Cd concentrations in the shoots were decreased, but significantly increased in the roots of *Solanum photeinocarpum* by *Glomus versiforme* colonization^21^. Moreover, Wu *et al.* also found that mycorrhizal inoculation remakably decreased As concentrations in husk, straw and root of upland rice grown in As-added soils (70 μg As g^−1^ soil)^14^. In the present study, Gv inoculation significantly reduced Cd concentrations in the roots and shoots, and Ri presence also evidently reduced Cd concentrations in the shoots but increased Cd concentrations in the roots of *L. japonica*. The reduced shoot Cd concentration in mycorrhizal *L. japonica* could be explained by the possible mechanisms (1) mycorrhizal hyphae can serve as a Cd pool to prevent Cd translocation to shoots by adsorbing and binding Cd^35^,^36^, and (2) the “dilution effects” linked to an increased plant biomass and a decreased Cd allocation to above-ground tissues^37^,^38^. In a word, both Gv and Ri colonization significantly reduced Cd concentrations in the shoots of *L. japonica*, which would provide a clue for safe production of this plant from Cd-contaminated soils.

SOD is involved in converting superoxide to H_2_O_2_, and CAT, POD and APX are mainly responsible for the dismutation of H_2_O_2_ to H_2_O and O_2_. Liu *et al.* reported that the activities of SOD, POD and CAT in the marigold inoculated with *R. intraradices* were higher than those of the uninoculation plants under Cd stress^39^. Similarly, Garg and Aggarwal observed that *Glomus mosseae* colonization significantly increased activities of SOD, CAT and POD in *Cajanus cajan* grown in the Cd and/or Pb contaminated soils^40^. Our previous experiment also indicated that *Solanum photeinocarpum* with *G. versiforme* and *S. nigrum* with *F. mosseae* had a higher activity of APX, POD and CAT than the uninoculation plants in Cd-added soils^21^,^28^. In the present study, the enhancement of CAT and APX activities in mycorrhizal plants suggested that both Gv and Ri colonization helped *L. japonica* to alleviate oxidative stress.

In antioxidative metabolisms, GSH, ASA and GR play an important role in removing H_2_O_2_ by the ascorbate-glutathione pathway^41^. The present studies exhibited that GR activity of mycorrhizal plant was increased at all Cd levels by comparing with non-mycorrhizal plant, which was similar with previous findings. For example, Garg and Kaur reported that GR activity had an increase in AMF-inoculated *C. cajan* under Cd and/or Zn stresses^8^. Garg and Aggarwal observed also that *G. mosseae* inoculation evidently enhanced GR activity in *C. cajan* grown in the Cd and/or Pb contaminated soils^40^. Moreover, the contents of GSH and ASA in *L. japonica* were affected by Gv/Ri symbiosis. These results indicated both Gv and Ri inoculation had an influence on the ascorbate-glutathione pathway in *L. japonica*.

Lipid peroxidation is initiated due to oxidative stress, and high MDA accumulation shows severe lipid peroxidation. In this work, the MDA contents in inoculated plants were obviously reduced compared with uninoculated plants at all Cd levels, which further showed that both Gv and Ri inoculation reduced the Cd-induced oxidative stress in *L. japonica*.

PCs are cysteine-rich peptides synthesized from GSH in the presence of metal ions, and they are involved in metal detoxification^42^. As far as we are aware, there are few reports about the impact of AMF on PCs production under HM stress, and the results are controversial in different studies. For example, Garg and Kaur found *G. mosseae* colonization evidently increased PCs contents in *C. cajan* under Cd and/or Zn stress^8^. However, our previous study indicated that PCs synthesis in *S. nigrum* grown in different Cd-amended soil was not affected by *F. mosseae* colonization^28^. In the present study, the augment of PCs contents in mycorrhizal plants inferred that Gv/Ri-inoculated *L. japonica* may be more effective in alleviating Cd toxicity. In addition, present study showed that the GSH contents in inoculated plants were reduce compared with uninoculated plants, which might be chiefly attributed to the improvement of PCs synthesis in mycorrhizal plants.

## Conclusions

In our present study, the impacts of both Gv and Ri symbiosis on Cd uptake and some physiological parameters of *L. japonica* planted in Cd-amended soils were investigated, and conclusions were made as follows: Firstly, both Gv and Ri inoculation greatly improved plant growth due to increasing P acquisition. Secondly, Gv inoculation significantly reduced Cd concentrations in the roots and shoots, and Ri presence also significantly reduced Cd concentrations in the shoots but increased Cd concentrations in the roots of *L. japonica*. The decrease of Cd concentrations in the shoots of Gv/Ri-inoculated *L. japonica* would provide a clue for safe production of this plant from Cd-contaminated soils. Finally, activities of CAT, APX and GR, and PCs production and ASA contents in inoculated plant were higher than those of uninoculated plant, but the lower GSH and MDA contents were measured in the inoculated plants. In order to further explore the mechanisms of Cd toxicity relieving by AMF, we will turn to the molecular biology and proteomics researches in AMF-inoculated *L. japonica* planted in Cd-contaminated soil.

## Methods

### Materials preparation

Loamy soils used in the experiment were as described by Liu *et al.*^43^, with the following characteristics: pH 6.85 (1:1 w/v water), organic content 1.65%, available P 52 μg g^−1^, total Cd 0.12 μg g^−1^ and DTPA-extractable Cd 0.063 μg g^−1^. The soil was sieved to pass a 2 mm mesh and autoclaved (121 °C, 2 h) to sterilization. Before use, the sterile soil was divided into three aliquots amended with 0 (control), 10, 20 μg Cd g^−1^ soil (supplied as CdCl_2_), respectively. At the same time, Cd-added soil was subjected to equilibrium with aseptic water saturating for one month and air drying for one month in a controlled greenhouse at 28/22 °C with 14/10 d/night.

*Glomus versiforme* (Gv) and *Rhizophagus intraradices* (Ri) obtained from the Beijing Academy of Agriculture and Forestry, China, were propagated by *Zea mays* as the host plant growing in 2-L pots containing a 1:1 (v/v) mixture of soil and sand. After five months, the roots were cut into pieces and evenly mixed with the culture medium including rhizosphere soil, hyphae and spores, and all of the mixtures were used as AMF inocula.

### Pot experiment

There were three Cd levels (0, 10, 20 μg Cd g^−1^soil) and three AMF inoculations (with Ri, with Gv and without AMF) in a full randomized design with five replicates per treatment for a total of 45 experimental units.

The soil (2.1 kg) mentioned above was loaded into each pot (height 14 cm, bottom diameter 13 cm and top diameter 16 cm). Inoculated treatment was implemented by mixing 85 g mycorrhizal inocula in each pot. Each pot of the non-mycorrhizal treatments received the same amount of autoclaved inocula (121 °C for 2 h) together with a 30-ml aliquot of a filtrate (11 μm) of the AM inoculum for adding the microbial population free of AM propagules^44^.

The seeds of *L. japonica* were sterilized with 10% NaClO for 10 min, and washed with sterile water, and then germinated on sterilized sand in light-controlled incubator at 20 °C with 16/8 d/night regime. Four uniform seedlings were transplanted to each pot and grown in a controlled greenhouse at 28/22 °C with 14/10 d/night, and 60% of the water holding capacity. Water loss was compensated with sterile water every day, after weighing pots.

### Sampling

After 4 months, all plants of each pot were separated into root and shoot after harvesting. Fresh leaves were lyophilized and kept in vacuum desiccators for the physiological measurement. Roots were immersed in 0.01 M ethylene diamine tetraacetic acid (EDTA) for 30 min, and then washed with deionized water to remove metal ions of root surface^45^. In addition, the rhizosphere soils were sampled for further analysis.

### Mycorrhizal colonization

Cuttings of cleaned roots (1 cm) were softened in 10% KOH (w/w) for 30 min at 90 °C Water-both, bleached in 10% H_2_O_2_ for 30 min and acidified in 1% HCl for 3 min at 24 °C. Subsequently, roots were stained with 0.05% Trypan Blue (w/w) at 90 °C for 30 min and kept in lactic acid-glycerol solution (v/v 1:1)^46^. Forty pieces of fine roots collected from each pot were analysed and the AMF colonization rate was calculated according to the grid-line intersect method of Giovannetti and Mosse^47^.

### Plant and soil analysis

The shoots and roots were weighed after drying at 80 °C for 3 days. The dried samples were measured Cd and P concentrations after ground and digested in a tri-acid mixture (5:1:1 HNO_3_:H_2_SO_4_:HClO_4_) at 225 °C for atomic absorption spectrophotometry (AAS) (Z-2000, Hitachi, Japan) and molybdenum–ascorbic acid spectrophotometry^48^, respectively. DTPA-extractable Cd concentrations in rhizosphere soils were measured using the methods described by Zan *et al.*^49^.

The SOD activity was determined based on SOD’s ability to inhibit the reduction of nitroblue tetrazolium (NBT) by 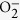 radical^50^. The APX activity was measured as the decrease in absorbance at 290 nm, according to APX’s ability to catalyze the oxidation of ascorbate^51^. The GR activity was measured by the reduction of absorbance at 340 nm due to NADPH oxidation^52^. The CAT activity was tested on the basis of the consumption rate of H_2_O_2_ at 240 nm^53^.

The ASA was measured according to the method of Law *et al.* using dipyridyl as the substrate^54^. MDA was tested as Heath and Packer described method by thiobarbituric acid (TBA) reaction^55^. GSH content was estimated by the method of O-Phthalaldehyde (OPA) fluorescence derivatization^56^. PCs content was measured as the difference between non-protein thiols (NPT) and GSH^57^. NPT content was assessed by the method of Ellman using 5,5′-dithiobis-(2-nitrobenzoic acid) (DTNB) as the substrate^58^.

### Statistical analysis

Data presented were means of five replicates, and appropriate transformations on the data were made prior to analysis to decrease the heterogeneity of the variance. The effects of mycorrhizal inoculation and Cd addition level and their interactions on measured variables were assessed by a two-way analysis of variance (ANOVA) at *p* < 0.05, 0.01, or 0.001. Means were compared using the Tukey test at *p* < 0.05. In all cases, statistical analyses were performed using the SPSS 17.0 (SPSS, Inc., Chicago, IL, USA).

## Additional Information

**How to cite this article**: Jiang, Q.-Y. *et al.* Can arbuscular mycorrhizal fungi reduce Cd uptake and alleviate Cd toxicity of *Lonicera japonica* grown in Cd-added soils? *Sci. Rep.* **6**, 21805; doi: 10.1038/srep21805 (2016).

## Figures and Tables

**Table 1:** Significance level of influences of different factors and factor interactions on variables based on two-way analyses of variance (ANOVA).

Variables	*F*-values		
	AMF inoculation	Cd concentration	Cd × AMF
Shoot biomass	28.17***	2.01 NS	2.14 NS
Root biomass	28.13***	4.86*	2.01 NS
Shoot P concentration	26.08***	48.67***	2.14 NS
Root P concentration	25.03***	20.35***	0.15 NS
Shoot Cd concentration	147.21***	33.94***	14.53**
Root Cd concentration	44.74***	105.38***	7.03*
Soil DTPA-Cd concentration	2.01 NS	797.05***	1.44 NS
SOD activity	0.81 NS	6.41**	2.21 NS
APX activity	803.67***	265.32***	51.48***
CAT activity	643.15***	306.03***	97.73***
GR activity	270.81***	382.01***	94.36***
MDA content	99.62***	97.73***	7.19**
ASA content	388.19***	21.93***	28.19***
GSH content	33.86***	53.93***	1.25 NS
PCs content	206.59***	212.95***	3.34*

CAT, APX, SOD, GR, GSH, ASA, MDA and PCs represent catalase, ascorbate peroxidase, superoxide dismutase, glutathione reductase, reduced glutathione, ascorbate, malonaldehyde and phytochelatins, respectively. NS – not significant; *significant at the level *p* < 0.05; **significant at the level *p* < 0.01; ***significant at the level *p* < 0.001.
